# An Integrative Transcriptome-Wide Analysis of Amyotrophic Lateral Sclerosis for the Identification of Potential Genetic Markers and Drug Candidates

**DOI:** 10.3390/ijms22063216

**Published:** 2021-03-22

**Authors:** Sungmin Park, Daeun Kim, Jaeseung Song, Jong Wha J. Joo

**Affiliations:** 1 Department of Computer Engineering, Dongguk University, Seoul 04620, Korea; trs9904@gmail.com; 2 Department of Life Science, Dongguk University, Seoul 04620, Korea; daeunkim115@gmail.com (D.K.); jaeseung6455@gmail.com (J.S.)

**Keywords:** amyotrophic lateral sclerosis, transcriptome-wide association study, drug repositioning, enrichment analysis, causal gene

## Abstract

Amyotrophic lateral sclerosis (ALS) is a neurodegenerative neuromuscular disease. Although genome-wide association studies (GWAS) have successfully identified many variants significantly associated with ALS, it is still difficult to characterize the underlying biological mechanisms inducing ALS. In this study, we performed a transcriptome-wide association study (TWAS) to identify disease-specific genes in ALS. Using the largest ALS GWAS summary statistic (n = 80,610), we identified seven novel genes using 19 tissue reference panels. We conducted a conditional analysis to verify the genes’ independence and to confirm that they are driven by genetically regulated expressions. Furthermore, we performed a TWAS-based enrichment analysis to highlight the association of important biological pathways, one in each of the four tissue reference panels. Finally, utilizing a connectivity map, a database of human cell expression profiles cultured with bioactive small molecules, we discovered functional associations between genes and drugs to identify 15 bioactive small molecules as potential drug candidates for ALS. We believe that, by integrating the largest ALS GWAS summary statistic with gene expression to identify new risk loci and causal genes, our study provides strong candidates for molecular basis experiments in ALS.

## 1. Introduction

Amyotrophic lateral sclerosis (ALS), also known as Lou Gehrig’s disease, affects nerve cells in the brain and spinal cord, causing motor neurons to mutate and disappear selectively and progressively. The disorder causes muscle weakness, such as myocardial lateral sclerosis and severe myocardial dysfunction, for which the causes are unclear and no definitive diagnostic tests exist so far [1]. The annual incidence of ALS is about 2 per 100,000 persons and the incidence rises considerably in people above mid-age [2].

Many studies have been conducted to identify the genetic mutations causing ALS, not only to understand the disease mechanisms but also to facilitate the design of disease modeling and treatment [3,4]. Although genome-wide association studies (GWAS) have successfully identified many variants significantly associated with ALS, it is often difficult to characterize the underlying biological mechanisms inducing ALS. Furthermore, the actual effects of loci may not completely be described because of complex factors such as linkage disequilibrium (LD) between the variants and differences in the amount of gene expression (GE) of particular genes in various tissues [5,6,7]. In order to understand the underlying biology, researchers have used expression quantitative trait loci (eQTL) studies, in which correlations between single nucleotide polymorphisms (SNPs) and GE have been studied to discover disease mechanisms at the gene level [8]. Unfortunately, not many expression-trait associations have been identified, given the sparsity of specimen availability and the prohibitive cost, especially for genes with small effect sizes.

In recent times, transcriptome-wide association study (TWAS) has emerged as a new technology to identify functionally relevant genes whose expression levels are associated with a trait of interest. Instead of measuring GE levels directly from samples, TWAS utilizes reference panels to learn a predictive model on an eQTL dataset and impute GE levels into a larger GWAS [9]. Using large-scale GWAS statistics, several TWAS successfully identified genes, even those with small effect sizes, that are significantly associated with complex traits, by increasing the statistical power in the analysis [10,11]. Several TWAS tools have been proposed, including FUSION, which is one of the representative tools [12,13,14]. To increase the power of analysis, FUSION provides reference panels to impute gene expression, which are used to calculate the causal relationship between SNPs and GE within specific tissues. In addition, in order to more accurately prioritize causal genes taking into account *cis*-genetic expression factors in GWAS summary statistics, FUSION analyzes by adding an LD matrix containing LD information between SNPs [15].

In this study, we used FUSION to perform TWAS on ALS and analyzed the largest ALS cohort ever to identify the candidate genes associated with ALS. The cohort consists of 20,806 ALS cases and 59,804 controls from Europe. Brain and blood-related panels from Genotype-Tissue Expression project v7 (GTEx v7) have been used as reference panels. From the analysis, we identified 27 significantly associated genes (20 genes are reported elsewhere and seven novel genes), showing that they are indirectly involved in the biological mechanisms of ALS [5,16,17]. Utilizing the LD information, we narrowed the 27 genes to 25 genes.

A conditional analysis is used to determine whether the significant TWAS gene has shown up owing to an independent association or a number of correlated single associations [18]. Through FUSION conditional analyses, which determine the independences of resulting genes, we demonstrate that several of the genome-wide significant signals from the ALS GWAS are driven by genetically regulated expression. Based on the TWAS-based gene set enrichment analysis (TWAS-GSEA), we confirmed the functional enrichment pathway of ALS. In addition, we performed drug repositioning analysis using significant TWAS genes using the connectivity map (CMap) software [19]. Finding effective treatment for ALS remains one of the biggest issues to solve. ALS is one of the most rapid neurodegenerative diseases, but so far, other than Riluzole, no drugs that slow disease progression have been reported, and numerous drugs have shown poor results in randomized controlled trials [20]. By means of the drug repositioning analysis using CMap, we detected 15 bioactive small molecules as potential drug candidates for ALS. This study is aimed at the comprehensive and in-depth characterization of the associations between cis-genetic components and ALS through comprehensive analysis in a multitude of relevant tissues using TWAS.

## 2. Results

### 2.1. Workflow of this Study

**Figure 1:**
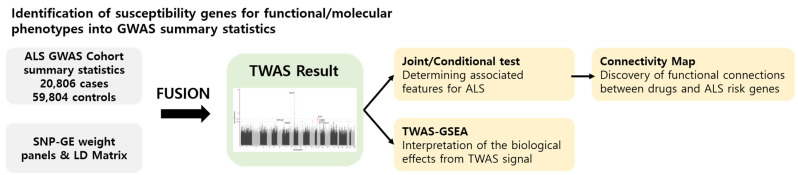
Analytical workflow.

To study the genetic mechanisms of ALS, a TWAS was performed using FUSION software. Figure 1 shows the workflow of our TWAS analysis, where ALS GWAS summary statistics, reference brain and blood tissue panels, and an LD matrix have been used as the input for FUSION software (http://gusevlab.org/projects/fusion/; accessed on 15 August 2020). The FUSION returns TWAS-identified transcriptome-wide significant genes responsible for ALS risk. After the analysis, conditional analyses were conducted on the significant TWAS genes to determine whether they were actually detected as independently significant associations. TWAS-GSEA was performed using significant TWAS genes to investigate the biological effects derived from the TWAS results and to explore biological pathways involved in the pathogenesis of ALS. Drug repositioning analysis was conducted in CMap using significant TWAS genes to discover drug candidates for ALS.

### 2.2. Identification of Significant TWAS Genes Associated with ALS Risk

**Figure 2:**
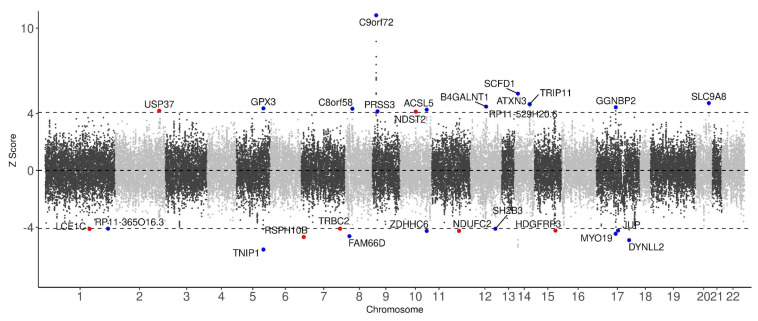
Manhattan plot of TWAS associations between gene expression and ALS. Each dot represents a TWAS signal. The x-axis corresponds to the chromosomal position of the genes and the y-axis corresponds to the Z-score value representing the predicted expression level of each gene in ALS. Black dashed lines indicate the transcriptome-wide significance threshold (FDR < 0.05). The names of statistically significant genes (FDR < 0.05) are labeled on the corresponding dots. Red and blue dots indicate novel risk genes and previously reported genes for ALS, respectively.

To identify risk genes contributing to the pathogenesis of ALS, we performed a TWAS using FUSION, with GWAS summary statistics of 20,806 ALS cases and 59,804 controls from Europe, 19 SNP-GE weight brain and blood panels from the GTEx consortium, and an LD matrix estimated from the 1000 Genomes Project. As ALS affects nerve cells mainly in the brain and other related organs, the 19 SNP-GE weight panels associated with brain tissue were selected for this study. All of the TWAS genes are listed in Supplementary Table S1. Applying a threshold of FDR < 0.05, 55 genes have been identified from 19 panels, of which 27 genes were uniquely identified (Figure 2 shows a Manhattan plot of TWAS associations between gene expression and ALS. Supplementary Table S2 lists 55 significant genes and their information including *p*-value and FDRs). Among the 55 TWAS genes, 35 were estimated to be up-regulated in ALS (Z-score > 0), whereas 20 to be down-regulated (Z-score < 0) (Supplementary Table S2). Twenty out of the 27 uniquely identified genes have been reported as ALS-associated genes in previous studies [5,21,22,23,24]. In particular, the top three of those 27 genes, i.e., *C9orf72-SMCR8 complex subunit* (*C9orf72*) (*p*-value = 1.65 × 10^−28^ and FDR = 4.03 × 10^−23^), *sec1 family domain containing 1 (SCFD1)* (*p*-value = 6.65 × 10^−8^ and FDR = 5.07 × 10^−4^), and *ataxin 3* (*ATXN3*) (*p*-value = 3.14 × 10^−6^ and FDR = 6.73 × 10^−3^), are well-known ALS-associated genes [5,16,17]. Table 1 lists the remaining 7 TWAS genes, which are, to the best of our knowledge, novel risk genes. The most significant among them is *radial spoke head 10 homolog B* (*RSPH10B*) (Z-score = −4.68, *p* = 2.90 × 10^−6^ and FDR = 6.43 × 10^−3^) from the pituitary gland. Among the brain tissues panels, the most significant novel gene found was *NADH:ubiquinone oxidoreductase subunit C2* (*NDUFC2*) (Z-score = −4.24, *p*-value = 2.18 × 10^−5^, and FDR = 3.04 × 10^−2^). Among the blood tissue panels, *hepatoma-derived growth factor, related protein 3* (*HDGFRP3*) (Z-score = −4.23, *p*-value = 2.39 × 10^−5^, and FDR = 3.24 × 10^−2^) was the most significant novel gene found.

**Figure 3:**
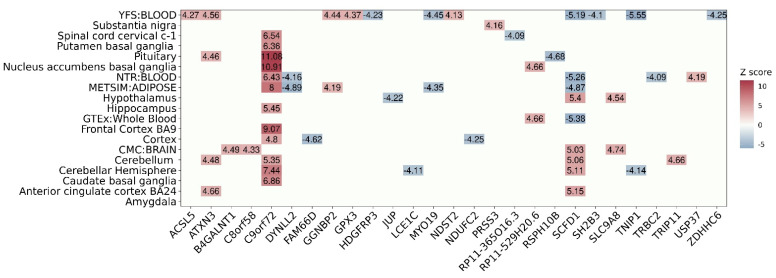
Heatmap showing the predicted expression patterns of TWAS significant associations. The x-axis represents the 27 significant TWAS genes and the y-axis indicates 19 SNP-GE weights. The Z-score values are labeled on the plot and are represented by a color bar on the right.

Next, we examined gene-tissue association from the 27 significant TWAS genes to assess the expression pattern of each gene in 19 SNP-GE weight panels (Figure 3 shows a heatmap representing the predicted expression patterns of TWAS significant associations). Of the 27 TWAS genes, 10 genes were associated with multiple tissue panels, while the other genes were detected from only one panel. Among the 10 genes, *C9orf72* was detected in 12 panels, the largest number of tissue panels among all genes. The expression levels of *C9orf72* were consistently estimated to be up-regulated in ALS patients in 12 panels. While most genes had consistent expression patterns across tissue panels, only *SCFD1* was predicted to be up-regulated by five SNP-GE panels associated with brain tissues and to be down-regulated by four SNP-GE panels associated with blood tissues in ALS patients. Consistent with our results, a previous study for ALS showed that the regression slope of *cis*-eQTLs of *SCFD1* was positive in brain tissues and negative in whole-blood tissues [23]. In terms of tissue panels, the SNP-GE weight panel associated with the largest number of genes, i.e., 11 TWAS associations, was the blood panel of YFS. Even though there can be interference in the tissue enrichment of TWAS associations because of different SNP-GE weight panels having different numbers of genetic features, we could suggest that this result is consistent with previous studies given that various metabolic biomarkers implicated in ALS pathogenesis have been detected in the blood [25,26]. To summarize, we identified 27 TWAS genes, including seven novel genes, as susceptibility genes contributing to the etiology of ALS and reported reliable gene-tissue associations in ALS. 

### 2.3. Conditional Analysis Supports the TWAS Genes of ALS

**Figure 4:**
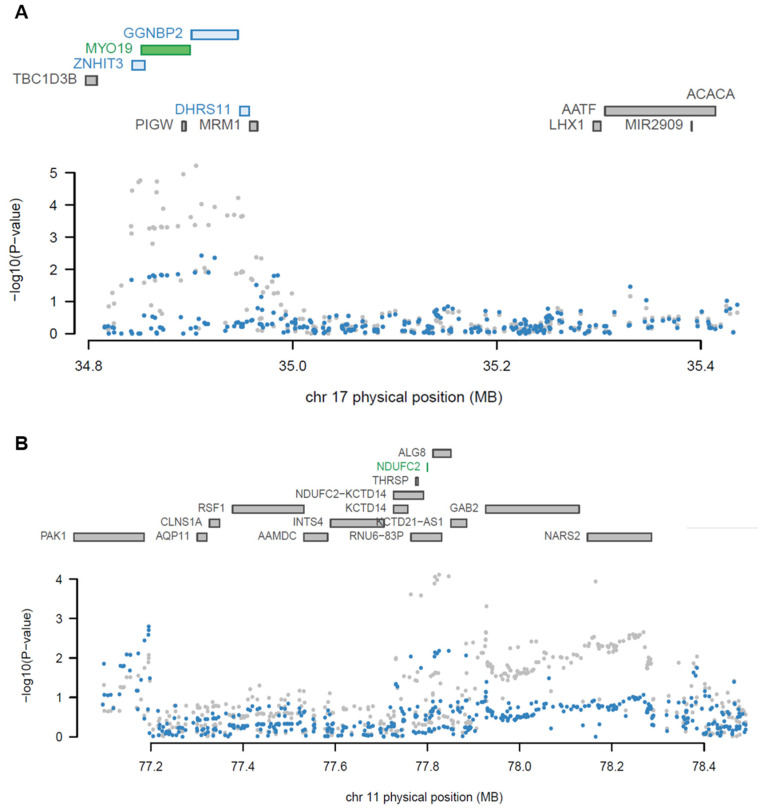
Regional association plots showing the jointly significant TWAS genes. (**A**) A regional association plot at chromosome 17 from blood tissue of YFS. (**B**) A regional association plot at chromosome 11 from the brain cortex of GTEx. Color bars on the top panel show all of the protein-coding genes and/or TWAS genes. Jointly significant genes are highlighted using green bars while marginally significant genes are highlighted using blue bars. A scatter plot at the bottom shows a Manhattan plot of the GWAS data before (gray) and after (blue) conditioning on the independently significant genes.

TWAS reports genes with significant causal relationships based on GWAS summary statistics. A conditional analysis using LD was performed to detect independent loci. The 27 TWAS genes were grouped into 22 different discrete loci by their physical position with a +/− 500 kb window (Supplementary Table S3 and S4). The result demonstrates that 25 of 27 significant TWAS genes were identified as independent TWAS signals at 22 loci, conditional on their predicted expression (conditional *p* < 0.05). *Myosin XIX* (*MYO19*) and *gametogenetin-binding protein 2* (*GGNBP2*) among the 27 TWAS genes, located at the same loci, were tested for the independence of significance. When conditioning on the predicted expression of *MYO19* in the blood panel, the GWAS signals significantly (lead SNP P_GWAS_ = 6.21 × 10^−6^ conditioned on *MYO19* lead SNP P_GWAS_ = 0.00369; Figure 4A) dropped. This result indicated that the expression of *MYO19* may explain most GWAS signals at the locus, implying that *MYO19* is a jointly significant gene (conditional *p*-value in blood of YFS = 8.6 × 10^−6^) but *GGNBP2* is not (conditional *p*-value in blood of YFS = 0.42). *NDUFC2*, one of the novel risk genes from brain tissue, was also identified as an independently significant gene, not being dependent on effects from GWAS SNPs, because the majority of GWAS signals were substantially altered after conditioning on the expression of *NDUFC2* in the brain cortex lead SNP P_GWAS_ = 7.85 × 10^−6^, conditioned on *NDUFC2* lead SNP P_GWAS_ = 1.61 × 10^−5^; Figure 4B). 

Furthermore, we compared 22 discrete loci containing 25 independent TWAS associations with GWAS loci reported by the GWAS catalog. Eight of the 22 loci were mapped within +/− 500 kb of the GWAS significant loci implicated in ALS Table 2. As expected, the abovementioned *C9orf73, SCFD1*, and *ATXN3* genes were simultaneously identified to be implicated in ALS at TWAS and GWAS significant loci. The majority of TWAS genes overlap the reported genes located at the mapped GWAS loci, implying that our TWAS results are reliable. Nevertheless, there are several TWAS genes that do not correspond to GWAS-reported genes at the overlapped loci, such as *ubiquitin-specific peptidase 37* (*USP37*), *serine protease 3* (*PRSS3*), *β-1,4-N-acetyl-galactosaminyltransferase 1* (*B4GALNT1*), and *MYO19*. One of the TWAS novel genes, *USP37*, was detected at significant GWAS loci and the other novel genes were not detected within +/− 500 kb of the GWAS loci. These results indicate that TWAS responsibly discovered risk genes for ALS that could not be detected by GWAS. Collectively, we identified that the majority of our TWAS genes were independently significant risk genes for ALS.

### 2.4. Functional Annotation of TWAS Signals

In order to explore the biological effects arising from the TWAS-identified genes of ALS, we conducted a TWAS-GSEA using TWAS signals and reference gene sets. TWAS-GSEA can analyze functional clusters of TWAS signals, minimizing the loss of signals from intermediating genes. TWAS signals were grouped by 19 different tissues and their functional categories were tested using reference gene sets, such as Hallmark and gene ontology (GO) gene sets. Four biological gene sets were significantly associated with TWAS signals in four tissue panels, i.e., hippocampus, hypothalamus, nucleus accumbens/basal ganglia, and whole-blood (FDR < 0.05) Table 3. Three gene sets, i.e., negative regulation of binding, perikaryon, and Golgi vesicle-mediated transport, were from GO, and a single gene set, KRAS signaling up, was from Hallmark. Four significantly enriched pathways contained a single TWAS significant gene (Supplementary Table S5). 

Negative regulation of binding was enriched in hippocampus tissue, which is consistent with previous reports of the correlation between ALS and cytoplasmic aggregation of RNA-binding protein [27]. Golgi vesicle-mediated transport was significantly enriched with TWAS genes in whole-blood tissue, which may indicate that the Golgi apparatus plays a crucial role in the pathogenesis of ALS [28,29,30]. *SCFD1*, one of the significant TWAS genes, is also involved in the endoplasmic reticulum (ER)-to-Golgi transport and was reportedly mutated in a small proportion of ALS patients [31]. Notably, up-regulation of the KRAS signaling pathway was enriched in two tissues, specifically, the hypothalamus and nucleus accumbens/basal ganglia, owing to the TWAS signal. In accordance with the importance of the KRAS pathway in several neuropathies, our result showed that dysregulation of the KRAS pathway might be also involved in the pathogenesis of ALS based on genetic features [32]. In addition, we identified that TWAS signals from the nucleus accumbens/basal ganglia were enriched in the perikaryon pathway. Neurofilament accumulation in the perikaryon was previously reported as one of the most common and characteristic pathological features of ALS [33,34]. We suggest that these four biological processes together might have a genetic contribution to the pathogenesis of ALS.

### 2.5. Identification of Drug Candidates for ALS

To discover drug candidates that can target the 27 TWAS genes, we conducted a drug repurposing analysis upon the TWAS results using the CMap software (Supplementary Table S6). CMap performs connectivity analysis of gene expression activation between bioactive small molecules by comparing the order of gene expression with the reference database (build 02). Table 4 lists 15 bioactive small molecules that were detected as potential drug candidates for ALS (*p* < 0.01). The bioactive molecule with the highest enrichment score is MK-886 (enrichment score = 0.966 and *p*-value = 2.01 × 10^−3^), which is known as an inhibitor of 5-lipoxygenase (5-LOX) and could thus reduce the development of atherosclerosis [35]. Klegeris et al. reported that 5-LOX inhibitors including MK-886 were identified as effective neuroprotective agents by suppressing toxic actions derived from reactive microglia that are associated with age-related degenerative diseases including ALS [35]. Trichostatin A (enrichment score = 0.386 and *p*-value = 0.00), the most statistically significant drug candidate, has shown protective effects in cell types involved in ALS through increased histone acetylation [36,37]. Yoo et al. suggested that trichostatin A may have a potential therapeutic effect to slow disease progression and to enhance motor performance in ALS patients [36].

The majority of other potential drug candidates that are identified in our analysis were reported to be either directly or indirectly associated with ALS elsewhere. STOCK1N-35696 (enrichment score = 0.965 and *p*-value = 2.07 × 10^−3^) was previously suggested as a potential therapeutic agent for Alzheimer’s disease [38]. Androsterone (enrichment score = 0.813 and *p*-value = 2.37 × 10^−3^) is a neurosteroid that could have effects on brain function, acting as a positive allosteric modulator of the γ-aminobutyric acid type A (GABA_A_) receptor [39,40]. Picrotoxinin (enrichment score = 0.801 and *p*-value = 3 × 10^−3^) is a component of picrotoxin that has been used as a stimulant of the central nervous system [41]. Deptropine is known as a classical histamine H1 receptor antagonist and histamine targets motor neurons, glial cells, and skeletal muscles, which all express histamine receptors, in ALS [42,43]. Volonté et al. reported that histaminergic modulation might be effective in ALS because histamine-related genes were dysregulated in the cortex and spinal cord in sporadic ALS patients [43]. Folic acid (enrichment score = 0.757 and *p*-value = 6.60 × 10^−3^) with vitamin B12 was reported to delay disease onset and prolong lifespans in the ALS mouse model [44]. Adenosine phosphate (enrichment score = 0.734 and *p*-value = 9.83 × 10^−3^) is involved in adenosine homeostasis that may reportedly play important roles in ALS progression [45]. Tetracycline (enrichment score = 0.673 and *p*-value = 9.75 × 10^−3^) is reportedly one of the antibiotics that could act as neuroprotective molecules in neurological diseases such as Huntington’s disease, Parkinson’s disease, stroke, and multiple sclerosis [46]. A previous study mentioned that phentolamine (enrichment score = 0.611 and *p*-value = 4.59 × 10^−3^) was effective to reduce blood pressure in a patient with ALS [47]. Collectively, we successfully detected 15 potential drug candidates associated with 27 TWAS genes, which may contribute to the development of new therapeutic options for ALS. 

## 3. Discussion

ALS is a disease that has been studied from various angles by several researchers. However, its exact biological mechanism remains unclear; consequently, there have been few definitive treatments for the disease. Although many genes, SNPs, and loci associated with ALS have been reported by GWAS studies, few studies have reported the functional significance of genetic components from a transcriptomic perspective. To overcome the limitation of GWAS studies, we performed a TWAS integrating the GWAS summary statistics of more than 80,000 ALS cohorts, 19 SNP-GE weights from brain and blood tissues, and the LD matrix. Since blood biomarkers have been steadily identified to explain various pathological mechanisms of ALS, we used SNP-GE weights of not only brain tissue but also blood tissue in order to provide new insights into the pathogenesis of ALS, compared with the previous ALS TWAS study [21]. Using the TWAS results, we conducted a functional enrichment analysis of TWAS signals and a drug repositioning analysis targeting significant TWAS genes. 

Twenty-seven significant TWAS genes were identified as susceptibility genes for ALS in 19 different SNP-GE weight panels related to the brain. Of the 27 significant TWAS genes, seven were novel risk genes identified by this study. Several novel genes can be biologically interpreted to be associated with ALS. *RSPH10B*, the most statistically significant TWAS gene, was reported to play a crucial role in ciliary and flagellar axonemes and to be associated with Kartagener syndrome [48]. Several genes responsible for Kartagener syndrome highlighted the importance of dynein motors to ciliary motility in a previous study [49]. Defects in cytoplasmic dynein-mediated retrograde axonal transport were reportedly implicated in the etiology of ALS [49]. Based on this information, we believe that *RSPH10B* may actually affect the pathogenesis of ALS and have a shared genetic contribution to Kartagener syndrome.

*NDUFC2,* the most significant novel gene from brain tissue, encodes a subunit of the mitochondrial membrane respiratory chain NADH dehydrogenase that is associated with mitochondrial diseases including Parkinson’s disease [50]. A previous study reported that the expression level of *NDUFC2* may contribute to the occurrence of ischemic stroke [51]. Another study on the aggregation of neurodegenerative disease proteins in cerebral ischemia revealed a previously unrecognized molecular overlap between neurodegenerative diseases such as ALS and ischemic stroke [51,52]. *USP37* encodes an enzyme that breaks down ubiquitin-specific processing proteins in the body [53]. Inhibition of *USP37* was reported to facilitate the proteasomal clearance of neurotoxic proteins [54]. Acting in a similar biological mechanism to *USP37*, *USP7* was suggested to be involved in the etiology of ALS associated with proteotoxicity [55]. In light of these facts, we suggest that our TWAS successfully identified biologically interpretable novel genes implicated in the pathogenesis of ALS.

Based on the conditional analysis, 25 out of 27 TWAS significant genes were identified as independent genes. We confirmed that the seven novel TWAS genes were located at different loci and were independently significant genes. Then, 25 genes were grouped into 22 discrete loci in which previously reported GWAS genes were implicated. Eight of the 22 loci were detected to simultaneously have TWAS- and GWAS-significant signals. *NDUFC2*, a novel risk gene not located at the eight loci, may not be detected by GWAS because of the small gene size and other large protein-coding genes nearby. However, TWAS identified *NDUFC2* as an independently significant gene, showing the power of TWAS to amplify weak signals associated with the disease. 

To investigate the biological effects derived from the ALS TWAS genes at the transcriptome level, we carried out a functional enrichment analysis using TWAS-GSEA based on the TWAS results. The TWAS signals were significantly enriched in 4 gene sets that were substantially consistent with those found in previous studies. Although the TWAS novel genes were not enriched in the four biological pathways, *junction plakoglobin* (*JUP*)*, C9orf72,* and *SCFD1*, which were previously reported as ALS-related genes, were included in the pathways (Supplementary Table S5). 

We also performed an in silico drug repurposing analysis using CMap to discover perturbations that can reverse the expression profiles of significant TWAS genes. CMap is a valuable approach to identify new indications for existing drug products and to potentially provide opportunities to develop new therapeutic options [56,57,58]. Fifteen bioactive molecules were detected as potential drug candidates for ALS using CMap, of which most molecules were directly or indirectly associated with ALS. Trichostatin A, the most statistically significant bioactive molecule associated with ALS, was reported to inhibit histone deacetylase 6 of which overexpression disrupted the localization of p58 mediating binding of Golgi elements to microtubules [59]. It may be consistent with the TWAS-GSEA results in terms of Golgi-related etiology for ALS. Even though the potential drug candidates were substantially implicated in ALS, functional studies may be necessary to validate their actual effects under physiological conditions because the results were derived from only in silico analyses. 

While the overall results from TWAS can be invaluable sources to understand the pathogenesis of ALS, there are several limitations to overcome. TWAS is a powerful approach to find risk loci affecting GE and to detect trait-associated genes; however, identifying the associations between genes and the trait does not explain the causality of disease. Thus, the genetic effects from dysregulation of significant TWAS genes need to be validated using further in vitro or in vivo studies. As the SNP-GE weight panels were used to predict the association of each eQTL and the disease in TWAS, the results of TWAS may depend on the number of features in the SNP-GE panels. Brain substantia nigra (n = 1604) and brain amygdala (n = 1837) panels have fewer features compared to the other tissue panels, which have more than 2000 features. In fact, no significant TWAS signals were identified in the brain amygdala tissue among 19 SNP-GE weight panels. Despite these limitations, we believe that our TWAS successfully identified biologically interpretable risk genes for ALS by contemplating the GE from the brain-related tissue panels. Our study can provide new biological insights into the pathogenesis of ALS and may contribute to the advancement of therapeutic options for ALS.

## 4. Materials and Methods

### 4.1. GWAS Summary Statistics of ALS

ALS GWAS summary statistics data was retrieved from the GWAS catalog (www.ebi.ac.uk, accessed on 15 August 2020; accession number: GCST005647). Details on participant ascertainment and quality control were described by Aude et al. [6]. The ALS GWAS used in this work includes only the European population (n = 20,806 cases and 59,804 controls). To use the GWAS summary statistics for TWAS analysis, the LD score (LDSC, v1.0.1) software was used to generate a sumstat-formatted file estimating SNP heritability and genetic covariance [15]. The generated file can correct the inflation values caused by GWAS, so the values predicted will be more accurate than in the original format.

### 4.2. Transcriptome-Wide Association Study

A TWAS was performed using FUSION software to conduct tissue-specific gene expression imputation based on the ALS GWAS summary statistics (http://gusevlab.org/projects/fusion/; accessed on 15 August 2020). TWAS incorporates ALS GWAS summary statistics into *cis*-eQTL information representing the relationship between SNPs and GE in specific tissues and accounts for LD to identify candidate genes associated with traits. The SNP-GE weights containing *cis*-eQTL information, which indicates the correlation between GE and SNP, were retrieved from the TWAS FUSION website. Four SNP-GE weights of blood tissue were obtained from four cohort datasets: Netherlands Twins Register (NTR), Young Finns Study (YFS), metabolic syndrome in men study (METSIM), and Genotype-Tissue Expression project v7 (GTEx v7, whole blood) [60,61,62,63]. Fifteen SNP-GE weights related to brain tissue were obtained from the Genotype-Tissue Expression project v7 (GTEx v7) and Common Mind Consortium (CMC). All SNP-GE weights are composed of genes being *cis*-regulated by SNPs within +/− 500kb of the transcription start site and having significant heritability measured using GCTA/REML with a *p*-value less than 0.01. An LD reference data for European populations from the 1000 Genomes project was used to contemplate the LD region [64]. FUSION calculates gene expression using a linear mixed model such as polygenic risk scores, LASSO, elastic net, and Bayesian sparse linear mixed model (BSLMM) [14,65]. It performs 5-fold cross-validation on all models to determine the best model and displays the optimized results. An FDR-corrected *p*-value < 0.05 was considered as a threshold for significant TWAS associations.

### 4.3. Conditional Analysis Using TWAS Results

To confirm whether the multiple association features are independent signals, a conditional analysis was conducted on the genomic regions where the identified significant TWAS genes are located, using FUSION software with the LD matrix [15]. The joint association *p*-value for each significant TWAS gene was calculated after conditioning on the expression of the gene. This analysis can be used to investigate whether the loci have truly independent associations when significant TWAS genes were identified at the loci using SNP-GE weights. Conditional analysis can also identify how much GWAS signal remains after the effects from the TWAS independent signal are removed.

### 4.4. Functional Enrichment Analysis

TWAS-GSEA was performed using TWAS results and SNP-GE weights to identify the enrichment of specific biological pathways in ALS [10]. All genes obtained from each tissue panel in TWAS were ranked using Z-scores and were used as pre-ranked gene sets, in order to account for even the smallest potential that may be missed by focusing only on significant genes. Gene sets of Hallmark and GO from molecular signatures database (MsigDB v7.1, http://software.broadinstitute.org/gsea/msigdb; accessed on 15 August 2020) were used as reference biological gene sets [66]. TWAS-GSEA utilizes a linear mixed model from the lme4qtl R package to test for an association between Z-scores and gene set membership while adjusting for non-biological effects and accounting for correlation between genes [67].

### 4.5. Drug Repositioning Analysis Using CMap

To discover drug candidates for ALS, drug repositioning analysis was conducted using CMap upon the TWAS genes statistically significantly associated with ALS. The formats of the TWAS genes were converted into the probe identifiers of Affymetrix Human genome U133a using the hgu133a.db Bioconductor annotation R package (“hgu133a2.db”). The TWAS genes were grouped as up- or down-regulated genes based on their Z-scores and the corresponding probe IDs of up- and down-regulated genes were respectively provided as down- and up-regulated genes to the CMap software. CMap (build 02) provides data on expression profiles of more than 7000 drug signatures representing 1309 bioactive compounds. The enrichment scores between CMap bioactive molecules and the genes provided as input were calculated based on the Kolmogorov–Smirnov statistical method. The range of enrichment score was represented between +1 and −1, indicating positive or negative enrichment between the bioactive molecules and the input genes. As the TWAS genes with opposite signs of their Z-scores were provided as the input of CMap, small molecules with a *p*-value < 0.01 and a positive enrichment score were selected as drug candidates for ALS.

## Figures and Tables

**Table 1:** Significant TWAS novel risk genes for ALS (FDR < 0.05). GTEx, Genotype-Tissue Expression project v7; YFS, Young Finns Study; NTR, Netherlands Twin Registry.

Gene	Tissue Panel	Chr	Best GWAS rsID	TWAS Z-Score	TWAS *p*-Value	FDR
*RSPH10B*	GTEx—Pituitary	7	rs709930	−4.68	2.90 × 10^−6^	6.43 × 10^−3^
*NDUFC2*	GTEx—Brain Cortex	11	rs665278	−4.25	2.18 × 10^−5^	3.04 × 10^−2^
*HDGFRP3*	YFS—Blood	15	rs13329195	−4.26	2.39 × 10^−5^	3.24 × 10^−2^
*USP37*	NTR—Blood	2	rs933994	4.20	2.74 × 10^−5^	3.54 × 10^−2^
*NDST2*	YFS—Blood	10	rs12256103	4.13	3.57 × 10^−5^	4.22 × 10^−2^
*LCE1C*	GTEx—Brain Cerebellar Hemisphere	1	rs3126091	−4.11	3.93 × 10^−5^	4.50 × 10^−2^
*TRBC2*	NTR—Blood	7	rs1964986	−4.09	4.28 × 10^−5^	4.83 × 10^−2^

**Table 2:** Independent TWAS genes mapped to GWAS loci reported by GWAS catalog.

Gene(s).	Chr	Gene Start Position	Gene Stop Position	GWAS Reported Gene	Index SNP	GWAS Loci Position
*USP37*	2	219,000,000	219,000,000	*STK36*, *TTLL4*, *ZNF142*	rs2303565	218,680,586
*GPX3*, *TNIP1*	5	150,000,000	150,000,000	*TNIP1*	rs10463311	15,103,274
*C9orf72*	9	27,500,000	27,600,000	*IFNK*, *MOBKL2B*, *C9orf72*	rs3849943, rs3849942	27,543,384
*PRSS3*	9	33,800,000	33,800,000	Intergenic	rs4879628	32,888,522
*B4GALNT1*	12	58,000,000	58,000,000	*KIF5A*	rs113247976	57,581,917
*ATXN3*, *TRIP11*, *RP11-529H20.6*	14	92,500,000	92,500,000	*ATXN3*	rs10143310	92,074,037
*SCFD1*	14	31,100,000	31,200,000	*SCFD1*	rs10139154	30,678,292
*MYO19*	17	34,851,477	34,900,737	*TMEM132E*	rs730547	34,785,087

**Table 3:** Biological processes significantly enriched with TWAS signals of ALS (FDR < 0.05). N mem avail denotes the number of TWAS signals involved in the reference gene set used in this analysis; N mem denotes the total number of genes in the reference gene set.

Panel	GeneSet	N Mem Avail	N Mem	FDR
Brain Hippocampus	GO: Negative regulation of binding	14	164	3.15 × 10^−2^
Brain Hypothalamus	Hallmark: KRAS signaling up	13	200	1.30 × 10^−2^
Brain Nucleus accumbens/basal ganglia	Hallmark: KRAS signaling up	14	200	2.40 × 10^−2^
	GO: Perikaryon	11	127	3.60 × 10^−2^
Whole-Blood	GO: Post Golgi vesicle-mediated transport	11	104	3.05 × 10^−2^

**Table 4:** Bioactive molecules discovered by CMap as potential drug candidates for ALS (*p* < 0.01).

CMap Name	PubChem Name	PubChem CID	Enrichment Score	*p*-Value
MK-886	MK 886	365137	0.966	2.01 × 10^−3^
STOCK1N-35696	-	-	0.965	2.07 × 10^−3^
PF-00539758-00	-	-	0.949	1.20 × 10^−4^
16,16-Dimethylprostaglandin E2	16,16-Dimethyl-Pge2	5283066	0.893	2.38 × 10^−3^
Bufexamac	Bufexamac	2466	0.827	1.35 × 10^−3^
Androsterone	Androsterone	5879	0.813	2.37 × 10^−3^
Picrotoxinin	Picrotoxinin	442292	0.801	3 × 10^−3^
Sulfinpyrazone	Sulfinpyrazone	5342	0.795	3.46 × 10^−3^
Deptropine	Deptropine	203911	0.77	5.43 × 10^−3^
Folic acid	Folic acid	135398658	0.757	6.60 × 10^−3^
Mebendazole	Mebendazole	4030	0.741	2.68 × 10^−3^
Adenosine phosphate	Adenosine 5’-Monophosphate	6083	0.734	9.83 × 10^−3^
Tetracycline	Tetracycline	54675776	0.673	9.75 × 10^−3^
Phentolamine	Phentolamine	5775	0.611	4.59 × 10^−3^
Trichostatin A	Trichostatin A	444732	0.386	0.00

## Data Availability

Data sharing not applicable.

## Supplementary Materials

The following are available online at https://www.mdpi.com/1422-0067/22/6/3216/s1, Table S1: TWAS summary statistics, Table S2: TWAS significant genes (FDR < 0.05), Table S3: Divide the loci of TWAS novel genes based on +/−500 kb, Table S4: Conditional analysis of multi-gene loci, Table S5: TWAS-GSEA result (FDR < 0.05) and enrichment gene set, Table S6: CMap permuted result.

## Abbreviations

ALS	Amyotrophic lateral sclerosis
GWAS	Genome-wide association studies
LD	Linkage disequilibrium
CMap	Connectivity map
eQTL	Expression quantitative trait loci
TWAS	Transcriptome-wide association study
GE	Gene expression
*C9orf72*	*C9orf72-SMCR8 complex subunit*
*SCFD1*	*Sec1 family domain* containing 1
*RSPH10B*	*Radial spoke head 10 homolog B*
*NDUFC2*	*NADH:ubiquinone oxidoreductase subunit C2*
*HDGFRP3*	*Hepatoma-derived growth factor, related protein 3*
*MYO19*	*Myosin XIX*
*GGNBP2*	*Gametogenetin-binding protein 2*
*USP37*	*Ubiquitin-specific peptidase 37*
*PRSS3*	*Serine Protease 3*
ER	Endoplasmic reticulum
*5-LOX*	*5-lipoxygenase*
GABA_A_	γ-aminobutyric acid type A
*JUP*	*Junction plakoglobin*
MsigDB	Molecular signatures database
